# Immunological mechanisms of low-grade systemic inflammation and its role in endometrial dysfunction in women with polycystic ovary syndrome

**DOI:** 10.3389/fimmu.2026.1846747

**Published:** 2026-07-09

**Authors:** Liuhua Qu, Lu Li, Ling Yang, Ying Liu

**Affiliations:** 1 Department of Central Laboratory, The Third Affiliated Hospital of Jinzhou Medical University, Jinzhou, Liaoning, China; 2 Department of Gynaecology, Ningyuan County People’s Hospital, Yongzhou, Hunan, China

**Keywords:** anti-inflammatory cytokines, endometrial receptivity, insulin resistance, low-grade systemic inflammation, polycystic ovary syndrome, pro-inflammatory cytokines, three-dimensional ultrasound blood flow parameters

## Abstract

**Background:**

Emerging evidence indicates impaired endometrial receptivity may contribute to reduced embryo implantation and poorer pregnancy outcomes in Polycystic ovary syndrome (PCOS) patients, though exact mechanisms are not yet fully elucidated.

**Objective:**

To explore the impact of low-grade systemic inflammation on endometrial dysfunction in women with PCOS, and to assess the relationship between inflammatory markers, endocrine and metabolic factors, and their link to endometrial receptivity.

**Methods:**

A retrospective analysis was conducted involving 180 infertile women with PCOS recruited from the gynecology department between January 2023 and June 2025 as the case group. The control group consisted of 180 women with regular menstrual cycles and no PCOS features, who experienced infertility due to male or tubal factors during the same period. Clinical data and metabolic/endocrine parameters, including the LH/FSH ratio, estradiol (E2), testosterone (T), and HOMA-IR, were collected. Serum levels of pro-inflammatory (IL-1β, IL-6, TNF-α) and anti-inflammatory (IL-4, IL-10) cytokines were quantified using ELISA. During the mid-luteal phase, transvaginal 3D ultrasound was used to assess endometrial thickness, pattern, and blood flow indices (VI, FI, VFI), as well as uterine artery Doppler parameters (PI, RI). Between-group differences were analyzed, along with correlations among inflammatory markers, metabolic profiles, and endometrial characteristics.

**Results:**

Compared to controls, the PCOS group had significantly higher BMI, LH/FSH ratio, T, and HOMA-IR (P<0.001). They showed increased pro-inflammatory cytokines (IL-1β, IL-6, TNF-α) and reduced anti-inflammatory cytokines (IL-4, IL-10) (P<0.001). Endometrial blood flow patterns were poorer, and uterine artery PI and RI were higher in the PCOS group (P<0.001), while endometrial thickness, VI, FI, and VFI did not differ significantly (P>0.05). Within PCOS group, pro-inflammatory cytokines correlated positively with BMI, LH/FSH ratio, T, HOMA-IR, and uterine artery PI and RI (r=0.44-0.58, P<0.001), whereas anti-inflammatory cytokines correlated negatively with these measures (r=-0.43 to -0.63, P<0.001). No significant correlations were observed with endometrial thickness or vascularization indices.

**Conclusion:**

Patients with PCOS exhibit state of low-grade systemic inflammation, characterized by elevated pro-inflammatory cytokines and reduced anti-inflammatory cytokines, which is closely associated with insulin resistance and hyperandrogenemia. This inflammatory state may contribute to development of endometrial dysfunction by increasing uterine artery blood flow resistance.

## 1 Introduction

Polycystic Ovary Syndrome (PCOS) is a prevalent endocrine-metabolic disorder affecting 5%-10% of women of reproductive age globally, with incidence rising annually (1). Characterized by ovulatory dysfunction, hyperandrogenism, and polycystic ovarian morphology, it is frequently associated with metabolic issues including obesity, insulin resistance, and hyperinsulinemia (2). Beyond impairing fertility, PCOS substantially elevates long-term risks for type 2 diabetes, cardiovascular disease, and endometrial cancer, posing a serious threat to reproductive health (3–6). For a long time, research on the mechanisms of PCOS-related infertility has mainly focused on abnormal follicular development and ovulatory dysfunction, with clinical treatment strategies centered on ovulation induction (7). However, accumulating evidence indicates that even after successful ovulation induction or the use of assisted reproductive technologies, the rates of embryo implantation, clinical pregnancy, and live birth in PCOS patients remain significantly lower than those in non-PCOS infertile women. This suggests that, in addition to oocyte factors, impaired endometrial receptivity is also a significant contributor to adverse pregnancy outcomes in PCOS patients (8, 9).

Endometrial receptivity refers to the special physiological state in which the endometrium allows for embryonic localization, adhesion, invasion, and ultimately implantation. Its establishment depends on the coordinated effects of a finely regulated hormonal network, the immune microenvironment, and local blood perfusion (10). During a normal menstrual cycle, the endometrium in the mid-luteal phase undergoes complex morphological and molecular changes under the sequential influence of estrogen and progesterone, preparing for embryo implantation. Recent years have seen notable international advances in the study of endometrial dysfunction associated with PCOS. Parker et al. (11) provided a comprehensive explanation of how metabolic and endocrine disturbances affect endometrial function at the molecular level. Their work highlights that hyperinsulinemia, insulin resistance, chronic low-grade inflammation, and hormonal imbalances—particularly in estrogen, progesterone, and androgens—jointly contribute to adverse alterations in the endometrial microenvironment. These effects are mediated through signaling pathways including PI3K/AKT/MAPK and Wnt/β-catenin, along with epigenetic regulatory mechanisms. These changes are closely associated with implantation failure, miscarriage, and pregnancy complications. This research also emphasized the central role of the synergistic interaction between inflammatory factors and hormonal signaling pathways in the pathophysiology of the PCOS endometrium. Research by Yang et al. (12), utilizing proteomic analysis, identified specific differences in protein expression in the endometrium of PCOS patients, such as ACTR1A and CKB. These proteins are correlated with adverse pregnancy outcomes, providing molecular-level evidence supporting the existence of abnormal endometrial function.

In addition to the aforementioned abnormalities in hormone signaling pathways, chronic low-grade inflammation has been recognized as one of the core pathophysiological features of PCOS. The study by Vasyukova et al. (13) indicates that chronic low-grade inflammation is an independent factor affecting PCOS and is not dependent on overweight or obesity. Unlike classical infectious inflammation, the low-grade systemic inflammation in PCOS patients is characterized by mildly elevated levels of pro-inflammatory cytokines in the circulation and a relative deficiency of anti-inflammatory factors, but without typical clinical signs of infection. This state of immune imbalance is closely related to the core metabolic abnormalities of PCOS. On one hand, obesity and insulin resistance can induce macrophage infiltration and polarization changes in adipose tissue, promoting the release of pro-inflammatory factors (14); on the other hand, hyperandrogenemia can directly activate immune cells, exacerbating the inflammatory response (15). Conversely, inflammatory factors can also exacerbate insulin resistance by interfering with insulin signal transduction pathways and promote excessive androgen secretion by upregulating the activity of ovarian local androgen synthesis enzymes, thus forming a vicious cycle (16).

As a target organ for embryo implantation, the endometrium’s responsive mechanism to systemic inflammatory status has garnered increasing attention. A complex immune cell network exists in normal endometrial tissue, including macrophages, natural killer cells, and T cell subsets. These cells undergo dynamic changes during the menstrual cycle, collectively maintaining an immune-tolerant microenvironment conducive to embryo implantation (17). When systemic low-grade inflammation occurs, pro-inflammatory factors in the circulation may act on endometrial vascular endothelial cells and stromal cells, leading to local microenvironmental disturbances (18). Studies have shown that chronic endometritis can impair endometrial receptivity through various mechanisms, such as inducing an imbalance in endometrial immune cell ratios, disrupting the hormonal regulatory network, and promoting abnormal angiogenesis (19). Although PCOS-related low-grade systemic inflammation and chronic endometritis have essential etiological differences, their impact on endometrial function may share common effector pathways. In particular, their regulatory effect on endometrial blood perfusion warrants in-depth investigation.

Although research on low-grade inflammation in PCOS is already extensive, most studies have focused on the associations between inflammation and metabolic abnormalities or between inflammation and endocrine disorders. However, systematic integrated analyses combining systemic inflammation, endocrine-metabolic indicators, and multidimensional parameters of endometrial receptivity remain rare. Based on this research background, this retrospective study enrolled infertile patients with PCOS as the study subjects, and use non-PCOS women with infertility due to male factor or tubal factor during the same period as controls. Peripheral blood inflammatory cytokine levels were systematically measured in both groups, along with the collection of endocrine-metabolic indicators and transvaginal three-dimensional ultrasound parameters of endometrial receptivity during the mid-luteal phase. By comparing the differences in various indicators, the correlation between systemic inflammatory markers and endocrine-metabolic indicators in PCOS patients was analyzed. Furthermore, the associations among inflammatory markers, endocrine-metabolic indicators, and endometrial receptivity parameters were explored. This study aims to provide clinical evidence for elucidating the immune-vascular mechanisms underlying PCOS-related infertility and to offer a theoretical basis for improving endometrial receptivity and reproductive outcomes in these patients.

## 2 Research methods

### 2.1 Study subjects

**Figure 1:**
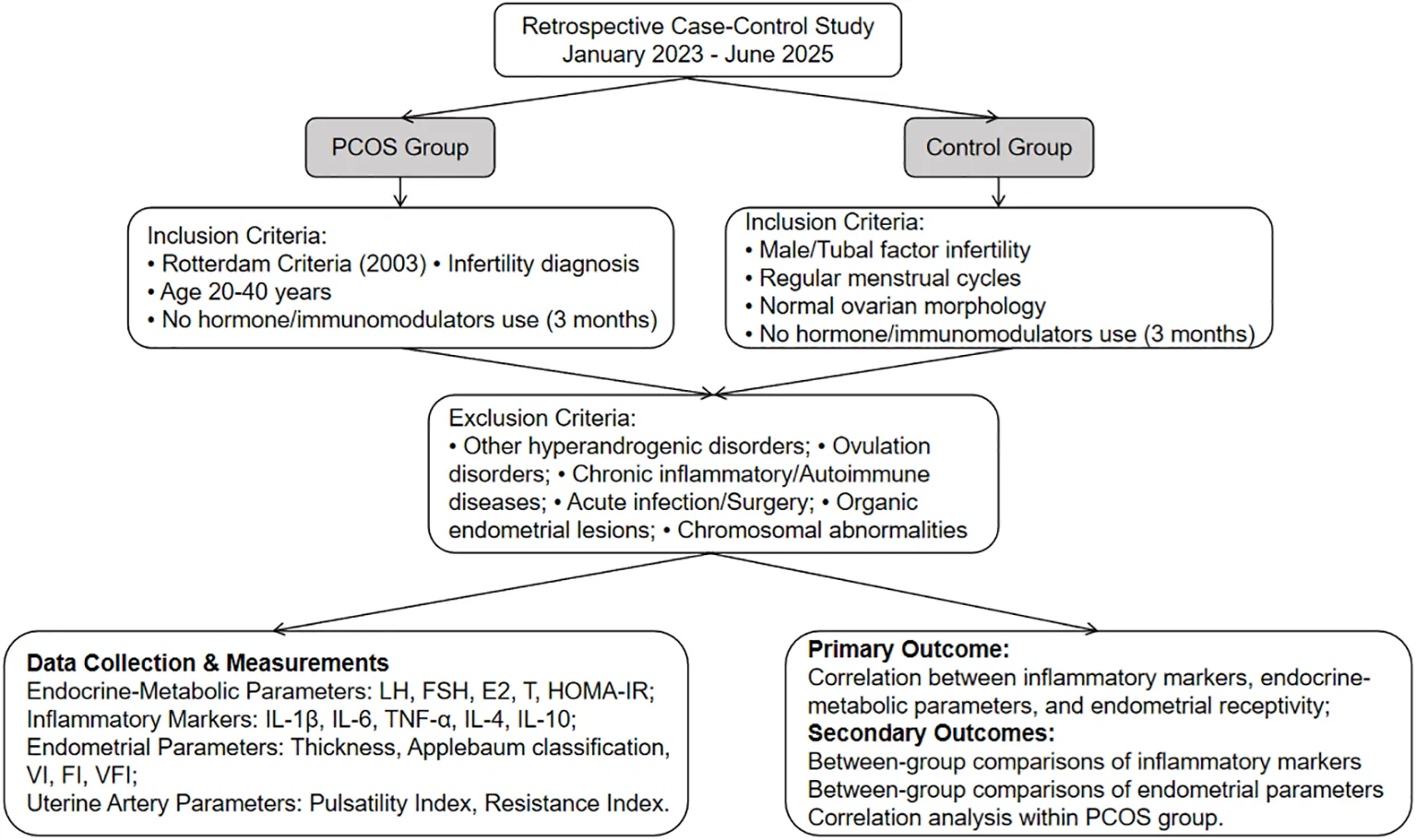
Research process.

This retrospective study was conducted in accordance with the principles of the Declaration of Helsinki. The study protocol was reviewed and approved by the Ethics Committee of Ningyuan County People’s Hospital (Approval No.【2026】-2). Due to the retrospective nature of the study and the use of anonymized clinical data, the requirement for informed consent was waived by the ethics committee. A total of 180 patients with PCOS complicated by infertility, who visited the gynecology department of our hospital for the first time between January 2023 and June 2025, were consecutively enrolled as the case group (PCOS group). In addition, 180 women who attended the center during the same period due to infertility caused by male factors or tubal factors, and who had regular menstrual cycles without any clinical manifestations or biochemical characteristics related to PCOS, were selected as the normal control group. The detailed study process is shown in **Figure 1**.

### 2.2 Inclusion and exclusion criteria

Inclusion Criteria for the PCOS Group: Diagnosis of PCOS based on the 2003 Rotterdam Criteria (20), updated according to the 2023 International Evidence-based PCOS Guidelines (2), requiring at least two of the following three criteria: ① Oligo-ovulation or anovulation; ② Clinical and/or biochemical hyperandrogenism; ③ Polycystic ovarian morphology (PCOM). Additionally, participants must meet the diagnostic criteria for infertility (excluding male factor infertility), be women of reproductive age between 20 and 40 years old, and have no history of using hormonal medications or drugs affecting immune function within the past three months.

Inclusion Criteria for the Control Group: Infertility due to male factors or fallopian tube factors; regular menstrual cycles with normal ovulation; ultrasound examination showing normal ovarian morphology without polycystic changes; no history of hormonal medications or immunomodulators within the past three months.

Common Exclusion Criteria for Both Groups: Conditions mimicking hyperandrogenism; disorders causing ovulatory dysfunction; chronic inflammatory or autoimmune diseases; acute infection or trauma/surgery within the prior month; organic uterine pathologies compromising endometrial receptivity; chromosomal abnormalities in either partner; and incomplete clinical data.

### 2.3 Sample size calculation

This study employed a case-control design, with the primary outcome being the difference in peripheral blood pro-inflammatory cytokine levels between the two groups. The sample size was estimated based on intergroup differences in tumor necrosis factor-alpha as reported in previous literature (21). With a statistical power (1-β) of 90% and a two-sided significance level of α=0.05, the required sample size was calculated to be approximately 158 cases per group. Ultimately, 180 participants were included in each group. This sample size also meets the requirements for statistical comparisons of secondary outcome measures, such as interleukin-6 and uterine artery blood flow perfusion parameters.

### 2.4 Research methods

1. Measurement of Endocrine and Metabolic Parameters: Peripheral venous blood (5 mL) was collected from all participants via fasting venipuncture during the follicular phase (days 3–5 of the menstrual cycle). Serum was isolated by centrifugation (3000 r/min for 10 min) and stored at -80 °C for batch analysis. Serum levels of luteinizing hormone (LH), follicle-stimulating hormone (FSH), estradiol (E2), and testosterone (T) were determined using a fully automated chemiluminescence immunoassay analyzer (Cobas e601, Roche Diagnostics, Switzerland) with original reagents, strictly following kit instructions. The LH/FSH ratio was calculated to assess ovarian function. The LH/FSH ratio was calculated by dividing the serum LH level (mIU/mL) by the serum FSH level (mIU/mL). Fasting plasma glucose (FPG) and fasting insulin (FINS) were also measured—FPG via the glucose oxidase method and FINS via chemiluminescence immunoassay. Insulin resistance was evaluated using the homeostasis model assessment (HOMA-IR), calculated as (FPG × FINS)/22.5, with HOMA-IR ≥ 2.69 indicating insulin resistance.

2. Systemic Inflammatory Marker Measurement: The protein expression levels of pro-inflammatory factors (IL-1β, IL-6, TNF-α) and anti-inflammatory factors (IL-4, IL-10) in peripheral blood serum were detected using enzyme-linked immunosorbent assay (ELISA). All ELISA kits used were purchased from R&D Systems (Minneapolis, USA).

3. Endometrial Receptivity-Related Parameter Measurement: All study participants underwent transvaginal three-dimensional ultrasound examination during the mid-luteal phase (7–9 days after ovulation). The instrument used was a color Doppler ultrasound diagnostic system (Model: Voluson E8, GE Healthcare, USA) equipped with a three-dimensional imaging probe (frequency: 5–9 MHz). The examination was performed by an experienced physician from the ultrasound department of our hospital, strictly adhering to ultrasound examination protocols. The measured parameters included: ① Endometrial thickness: The thickest part of the endometrium was selected on the ultrasound image, measured three times, and the average value was calculated, accurate to 0.1 mm. ② Endometrial pattern (Applebaum classification) (22): Classified into Type I, Type II, and Type III based on the ultrasound morphology of the endometrium. Type I (no intimal blood flow): color Doppler signal only reaches the outer myometrial layer (zone 1) without penetrating the subendometrial zone or endometrium, indicating poor vascularity; Type II (subintimal blood flow): color Doppler signal reaches the subendometrial zone (zone 2) but does not enter the endometrial layer; Type III (intimal blood flow): color Doppler signal penetrates into the endometrial layer (zone 3), reflecting good vascular perfusion. ③ Endometrial blood flow parameters: Using three-dimensional color Doppler ultrasound imaging, the vascularization index (VI), flow index (FI), and vascularization flow index (VFI) of the endometrium were measured. Each parameter was measured three times, and the average value was calculated. ④ Uterine artery blood perfusion parameters: The uterine artery pulsatility index (PI) and resistance index (RI) were measured. The segment of the uterine artery near the uterine myometrium was selected, and the PI and RI values were measured over three consecutive cardiac cycles, with the average value calculated.

### 2.5 Observation indicators and outcome definitions

The primary outcome measure of this study is the correlation between inflammatory markers, endocrine and metabolic indicators, and endometrial receptivity parameters. The secondary outcome measures are: Differences in peripheral blood inflammatory markers (IL-1β, IL-6, TNF-α, IL-4, IL-10) between the two study groups; Differences in endometrial receptivity-related parameters (endometrial thickness, typing, blood flow parameters, uterine artery blood flow parameters) between the two study groups; Correlations between inflammatory markers and endocrine and metabolic indicators (BMI, LH/FSH ratio, T, HOMA-IR) within the PCOS group.

### 2.6 Statistical analysis

Statistical analysis used SPSS 26.0. Normality was assessed via Shapiro-Wilk. Normally distributed data are presented as mean ± SD (t-tests), non-normal as median (Q1, Q3) (Mann-Whitney U). Categorical data are n (%) (χ² test). In the PCOS group, Pearson or Spearman correlation analyzed relationships among inflammatory, endocrine, and endometrial parameters. All tests were two-tailed, α=0.05.

## 3 Results

### 3.1 General clinical data and endocrine metabolic indicators

There was no statistically significant difference in age between the two groups (P>0.05), indicating comparability. Compared with the control group, the PCOS group showed significantly higher BMI, LH/FSH ratio, T level, and HOMA-IR, with statistically significant differences (P<0.001). However, there was no significant difference in E2 levels between the two groups (P>0.05). See **Table 1**.

**Table 1:** General clinical data and endocrine-metabolic parameters.

Indicators	Control group (n=180)	PCOS group (n=180)	Statistics	P
Age (years)	29.51 ± 3.24	29.81 ± 3.52	*t* = -0.856	0.392
BMI (kg/m²)	21.82 ± 2.51	24.93 ± 3.84	*t* = -9.099	<0.001
LH/FSH ratio	1.02 ± 0.25	1.89 ± 0.45	*t* = -22.605	<0.001
E2 (pg/mL)	48.57 ± 12.35	50.12 ± 14.56	*t* = -1.083	0.280
T (ng/dL)	50.12 ± 14.56	72.55 ± 15.83	*t* = -13.997	<0.001
HOMA-IR	1.65 (1.09, 2.04)	3.02 (2.25, 3.94)	*Z* = -12.168	<0.001

### 3.2 Comparison of systemic inflammatory marker levels between the two study groups

The results showed that the levels of pro-inflammatory cytokines IL-1β, IL-6, and TNF-α in the PCOS group were higher than those in the control group (P<0.001), while the levels of anti-inflammatory cytokines IL-4 and IL-10 were lower than those in the control group (P<0.001). In addition, the pro−/anti−inflammatory cytokine ratios were significantly different between the two groups. The median (Q1, Q3) IL−6/IL−10 ratio was 0.46 (0.34, 0.70) in controls versus 1.11 (0.72, 1.80) in the PCOS group (Z = 11.187, P<0.001). The finding further confirm a pronounced shift toward a pro−inflammatory state in PCOS patients. See **Table 2**.

**Table 2:** Serum inflammatory cytokine levels.

Parameters (pg/mL)	Control group (n=180)	PCOS group (n=180)	Statistics	P
IL-1β	3.02 (2.25, 3.94)	6.68 (5.46, 8.09)	*Z* = -14.335	<0.001
IL-6	4.58 (3.50, 5.60)	8.82 (6.91, 10.91)	*Z* = -13.645	<0.001
TNF-α	8.77 ± 2.81	15.62 ± 4.53	*t* = -17.262	<0.001
IL-4	6.69 (5.30, 8.35)	5.14 (3.62, 6.31)	*Z* = 7.409	<0.001
IL-10	10.04 (7.50, 12.00)	7.89 (5.78, 9.88)	*Z* = 5.180	<0.001
IL-6/IL-10	0.46 (0.34, 0.70)	1.11 (0.72, 1.80)	Z=11.187	<0.001

### 3.3 Comparison of endometrial receptivity parameters between the two study groups

The results of transvaginal three-dimensional ultrasound showed no statistically significant differences between the two groups in terms of endometrial thickness and three-dimensional power Doppler parameters (VI, FI, VFI) (P>0.05). However, the endometrial blood flow grading (Applebaum classification) in the PCOS group was significantly lower than that in the control group (P<0.001), manifested as an increased proportion of type I blood flow and a decreased proportion of type III blood flow. In addition, the uterine artery PI and RI in the PCOS group were significantly higher than those in the control group (P<0.001), suggesting increased uterine artery blood flow resistance and impaired uterine perfusion in patients with PCOS. See **Tables 3** and **4**.

**Table 3:** Endometrial thickness, 3D-PDA parameters, and uterine artery blood flow parameters.

Parameters	Control group (n=180)	PCOS group (n=180)	Statistics	P
Thickness (mm)	10.85 ± 1.95	10.62 ± 2.08	*t* = 1.085	0.279
3D-PDA Parameters				
Endometrial VI (%)	0.85 (0.55, 1.18)	0.79 (0.54, 1.14)	*Z* = 1.036	0.300
Endometrial FI	32.12 ± 5.63	31.69 ± 6.85	*t* = 0.636	0.525
Endometrial VFI	0.27 (0.19, 0.35)	0.25 (0.15, 0.35)	*Z* = 1.601	0.109
Uterine Artery Blood Flow Parameters				
PI	1.82 ± 0.42	2.31 ± 0.58	*t* = -8.940	<0.001
RI	0.79 ± 0.08	0.86 ± 0.09	*t* = -8.427	<0.001

**Table 4:** Endometrial blood flow classification (Applebaum classification) [n (%)].

Blood flow classification	Control group (n=180)	PCOS group (n=180)	Statistics	P
Type I (no intimal blood flow)	36 (20.00)	58 (32.22)	*Z* = 3.397	<0.001*
Type II (subintimal blood flow)	78 (43.33)	82 (45.56)		
Type III (intimal blood flow)	66 (36.67)	40 (22.22)		

*The P value indicates the overall difference in the distribution of the three endometrial blood flow types between the two groups (Mann-Whitney U test).

### 3.4 Correlation analysis between inflammatory factors and endocrine metabolic parameters in the PCOS group

In the PCOS group, pro-inflammatory factors (IL-1β, IL-6, TNF-α) were significantly positively correlated with BMI, LH/FSH ratio, T, and HOMA-IR (r=0.44-0.57, P<0.001). In contrast, anti-inflammatory factors (IL-4, IL-10) were significantly negatively correlated with the aforementioned parameters (r=-0.43 to -0.56, P<0.001). Furthermore, the calculated pro−/anti−inflammatory cytokine ratios were also significantly correlated with these metabolic and endocrine parameters. Specifically, the IL−6/IL−10 ratio exhibited even stronger positive correlations with BMI (r=0.578, P<0.001), LH/FSH ratio (r=0.540, P<0.001), T (r= 0.511, P<0.001), and HOMA−IR (r=0.589, P<0.001). These findings suggest that inflammatory status is closely associated with metabolic and endocrine disturbances. See **Table 5**.

**Table 5:** Correlation analysis between inflammatory cytokines and endocrine-metabolic parameters in the PCOS group.

Parameters		IL-1β	IL-6	TNF-α	IL-4	IL-10	IL-6/IL-10
BMI	r	0.531	0.566	0.508	-0.434	-0.514	0.578
	*P*	<0.001	<0.001	<0.001	<0.001	<0.001	<0.001
LH/FSH ratio	r	0.440	0.513	0.516	-0.497	-0.493	0.540
	*P*	<0.001	<0.001	<0.001	<0.001	<0.001	<0.001
T	r	0.508	0.567	0.568	-0.526	-0.513	0.511
	*P*	<0.001	<0.001	<0.001	<0.001	<0.001	<0.001
HOMA-IR	r	0.501	0.547	0.563	-0.449	-0.557	0.589
	*P*	<0.001	<0.001	<0.001	<0.001	<0.001	<0.001

### 3.5 Correlation analysis between inflammatory factors and endometrial receptivity parameters within the PCOS group

Pro-inflammatory factors (IL-1β, IL-6, TNF-α) showed a significant positive correlation with uterine artery PI and RI (r=0.45~0.58, P<0.001), but no significant correlation with endometrial thickness and VI, FI, VFI (P>0.05). Anti-inflammatory factors (IL-4, IL-10) were significantly negatively correlated with PI and RI (r=-0.51~-0.63, P<0.001). This suggests that inflammatory factors may impair endometrial receptivity by influencing uterine artery blood flow resistance. When examining the cytokine ratios, the IL−6/IL−10 ratio correlated positively with uterine artery PI (r=0.614, P<0.001) and RI (r=0.641, P<0.001) in the PCOS group. No significant correlations were found between the cytokine ratios and endometrial thickness or VI/FI/VFI. See **Table 6**.

**Table 6:** Correlation analysis between inflammatory cytokines and endometrial receptivity parameters in the PCOS group.

Parameters		IL-1β	IL-6	TNF-α	IL-4	IL-10	IL-6/IL-10
Thickness	r	-0.031	-0.050	-0.045	0.032	-0.060	0.002
	*P*	0.680	0.505	0.546	0.671	0.423	0.977
VI	r	-0.060	0.048	0.077	-0.043	-0.057	0.053
	*P*	0.425	0.523	0.304	0.566	0.448	0.478
FI	r	-0.034	-0.098	0.03	-0.013	-0.019	-0.025
	*P*	0.647	0.190	0.691	0.863	0.800	0.738
VFI	r	0.112	0.043	0.123	0.006	-0.133	0.100
	*P*	0.133	0.565	0.101	0.939	0.075	0.182
PI	r	0.569	0.576	0.554	-0.601	-0.575	0.614
	*P*	<0.001	<0.001	<0.001	<0.001	<0.001	<0.001
RI	r	0.445	0.545	0.501	-0.507	-0.628	0.641
	*P*	<0.001	<0.001	<0.001	<0.001	<0.001	<0.001

### 3.6 Correlation analysis between endocrine and metabolic indicators and endometrial receptivity parameters in the PCOS group

BMI, LH/FSH ratio, T, and HOMA-IR showed significant positive correlations with uterine artery PI and RI (r=0.43-0.67, P<0.001), but no significant correlations with endometrial thickness or VI, FI, and VFI (P>0.05). This suggests that metabolic and endocrine disorders may indirectly affect endometrial receptivity by increasing uterine artery blood flow resistance. See **Table 7**.

**Table 7:** Correlation analysis between endocrine-metabolic parameters and endometrial receptivity parameters in the PCOS group.

Parameters		BMI	LH/FSH ratio	T	HOMA-IR
Thickness	r	-0.030	0.029	0.028	0.008
	*P*	0.691	0.695	0.707	0.914
VI	r	0.023	-0.095	-0.015	0.063
	*P*	0.761	0.205	0.843	0.399
FI	r	-0.012	-0.032	-0.003	-0.018
	*P*	0.878	0.666	0.966	0.813
VFI	r	0.08	0.107	0.089	0.124
	*P*	0.286	0.151	0.235	0.098
PI	r	0.568	0.674	0.614	0.530
	*P*	<0.001	<0.001	<0.001	<0.001
RI	r	0.426	0.446	0.459	0.486
	*P*	<0.001	<0.001	<0.001	<0.001

## 4 Discussion

PCOS, as a prevalent endocrine and metabolic disorder, exerts effects that extend far beyond ovarian dysfunction and has increasingly been recognized in recent years as a chronic inflammatory state involving multiple systems throughout the body. Although previous studies have fully established the central role of insulin resistance and hyperandrogenism in the pathogenesis of this disease, the specific immunological mechanisms by which low-grade systemic inflammation participates in and exacerbates endometrial dysfunction, subsequently leading to reduced embryo implantation rates and adverse pregnancy outcomes, still require further elucidation (23). In this study, by systematically detecting the peripheral blood inflammatory cytokine profile, endocrine and metabolic parameters, as well as three-dimensional ultrasound parameters related to endometrial receptivity during the mid-luteal phase in patients with PCOS complicated by infertility, we aimed to clinically reveal the role of low-grade systemic inflammation in the regulation of endometrial function. The results showed that patients with PCOS exhibited a significant imbalance between pro-inflammatory and anti-inflammatory states. Moreover, this immune dysregulation was not only closely intertwined with classic metabolic and endocrine abnormalities but also demonstrated a significant association with resistance parameters reflecting uterine blood perfusion, independent of morphological changes in the endometrium. These findings provide new clinical evidence for understanding the pathophysiological processes underlying PCOS-related infertility.

This study reports that individuals with PCOS display a persistent, low-grade systemic inflammatory condition, marked by notably increased concentrations of pro-inflammatory cytokines IL-1β, IL-6, and TNF-α in peripheral blood, coupled with a relative lack of anti-inflammatory cytokines IL-4 and IL-10. These results align closely with current insights into the immunological dysregulation underlying PCOS. In their review, Parker et al. (11) systematically elaborated on the perspective that chronic low-grade inflammation is a core pathophysiological feature of PCOS, noting that elevated inflammatory cytokines such as TNF-α and IL-6 can directly disrupt the molecular microenvironment of the endometrium by interfering with local hormone receptor signaling pathways. Notably, a proteomics study by Butler et al. (24) on the complement system revealed that even non-obese PCOS patients exhibit significant upregulation of the alternative complement pathway, manifested by elevated levels of C3, C3adesArg, and C3a, and that these complement activation fragments are closely associated with the release of inflammatory cytokines. The study further indicated that activation of the complement pathway is an intrinsic characteristic of PCOS pathophysiology, with obesity merely exacerbating it rather than being the initiating factor. The results of the present study support the above viewpoint from a clinical data perspective and further reveal that the imbalance between pro-inflammatory and anti-inflammatory factors is a synergistic manifestation of systemic immune dysfunction in PCOS. A large cohort study by Jiang et al. (25) focusing on PCOS women undergoing *in vitro* fertilization also confirmed that inflammatory markers, represented by white blood cell count, are significantly correlated with adverse embryonic development outcomes, and that the inflammatory response partially mediates the negative impact of lipid metabolism abnormalities on embryo quality. This suggests that the upstream mechanism of the observed inflammatory cytokine imbalance in PCOS patients may involve aberrant activation of more fundamental immune and metabolic regulatory networks, such as the complement system, providing new insights for future exploration of intervention targets for inflammation in PCOS.

Correlation analysis showed that increased pro-inflammatory and decreased anti-inflammatory factors in PCOS patients were significantly associated with insulin resistance index and testosterone levels, indicating a complex bidirectional link between immune imbalance and metabolic-endocrine disturbances. On one hand, hyperinsulinemia in the context of insulin resistance can directly stimulate ovarian and adrenal androgen synthesis and may exacerbate systemic inflammatory responses by activating adipose tissue macrophages toward a pro-inflammatory phenotype (26). On the other hand, elevated pro-inflammatory factors such as TNF-α can further aggravate peripheral insulin resistance by inhibiting insulin receptor substrate signaling, thereby forming a self-reinforcing vicious cycle (27). A systematic review by Bai et al. (28) on endometrial receptivity also clearly indicated that altered expression levels of hormones and related receptors, an imbalance in cytokine ratios, as well as insulin resistance and chronic low-grade inflammation in PCOS patients, collectively constitute a multifaceted pathological basis that impairs endometrial receptivity. The findings of this study quantitatively reinforce this understanding, confirming a high degree of consistency between immune and metabolic parameters in PCOS patients. This suggests that therapeutic strategies for PCOS should simultaneously address both anti-inflammatory and metabolic improvement goals, rather than targeting a single component in isolation. This is best achieved by implementing lifestyle strategies (diet, exercise, behavioural) as is recommended for first-line treatment of all women with PCOS in the International Guidelines for the management of PCOS (29).

This study found that the peripheral blood inflammatory factor profile in patients with PCOS showed no significant correlation with either endometrial thickness or vascularization index of the endometrial layer during the mid-luteal phase. However, it exhibited a strong positive correlation with the pulsatility index and resistance index of the uterine arteries. This result suggests that low-grade systemic inflammation may not primarily impair endometrial receptivity by affecting endometrial thickness or local microvascular density. Instead, it may act on the uterine arteries, increasing their blood flow resistance, thereby reducing overall uterine blood perfusion at a macroscopic level. Research by Shan et al. (30), utilizing transcriptomic and epigenetic analyses of endometrial tissue samples from PCOS patients, revealed excessive ERα expression and histone lactylation modifications in the endometrium of these patients. Such molecular abnormalities are directly linked to decreased endometrial receptivity, indicating an inherent defect in the embryonic implantation microenvironment itself. Furthermore, the study by Liao et al. (31) confirmed at the molecular mechanism level that the downregulation of the IL-22-STAT3 signaling pathway and the subsequent inhibition of downstream IGFBP5 expression are significant causes of impaired endometrial receptivity in PCOS. Integrating the results of this study with these findings allows us to outline a more comprehensive pathological picture of systemic immune dysregulation. It may not only cause detrimental changes in the local microenvironment at the cellular and molecular level through pathways like PI3K/AKT/MAPK, as described by Parker et al. (11), but also reduce the delivery of key signaling molecules, such as IL-22, and essential nutrients to the endometrium via the hemodynamic pathway of increased uterine artery resistance. This synergistic effect, operating at both molecular and macro-hemodynamic levels, ultimately contributes to endometrial dysfunction. Of clinical relevance, elevated uterine artery PI is recognized as a predictive biomarker for subsequent pregnancy complications such as preeclampsia and fetal growth restriction (32), and these complications are 2–4 times more common in women with PCOS than in non-PCOS women (33).

Under chronic inflammatory conditions, elevated circulating factors such as TNF-α and IL-1β can act on vascular endothelial cells, leading to endothelial dysfunction. This is characterized by reduced bioavailability of nitric oxide and relatively enhanced activity of vasoconstrictors such as endothelin, resulting in impaired vasodilation and increased basal vascular tone (34, 35). In an early study by Piltonen et al. (36) investigating different cell types in the endometrium of women with PCOS, multiple cell populations—including epithelial cells, stromal fibroblasts, and mesenchymal stem cells—exhibited altered expression profiles of pro-inflammatory genes, suggesting that the entire uterine tissue is immersed in a pathological inflammatory microenvironment. This localized tissue inflammatory state may also affect the vascular wall of the uterine artery and spiral arterioles, inducing proliferation or phenotypic switching of vascular smooth muscle cells, ultimately leading to decreased vascular compliance and increased blood flow resistance. The study by Ampey et al. (37) provides important molecular evidence for understanding this phenomenon, revealing that tumor necrosis factor-alpha can directly impair the monolayer integrity of uterine artery endothelial cells and disrupt intercellular junction complexes via activation of the Src and ERK signaling pathways, thereby leading to vascular endothelial dysfunction and increased blood flow resistance. This finding directly elucidates how elevated circulating pro-inflammatory factors act on the uterine artery wall, contributing to the hemodynamic changes observed in the present study. Therefore, the increased uterine artery resistance observed in this study can be viewed as a functional response of the systemic and local vasculature to chronic low-grade inflammation.

This study also observed that the endometrial blood flow pattern in patients with PCOS was significantly poorer than that in the control group, primarily manifested as a significantly higher proportion of the poor blood flow pattern Type I, while the proportion of Type III blood flow, which represents good perfusion of the endometrium and subendometrial region, was significantly lower. Although the three-dimensional power Doppler parameters VI, FI, and VFI showed no significant differences, the difference in blood flow patterns, combined with elevated uterine artery resistance, still suggests a qualitative change in the uterine blood flow perfusion pattern in PCOS patients. This result may reflect the impact of inflammatory factors on the fine regulation of the vascular network at the endometrial-myometrial interface. Although parameters such as VI did not show differences in this study—possibly related to the sensitivity of the detection technique, the heterogeneity of the PCOS patient population, or sample size—the difference in blood flow patterns, together with elevated uterine artery resistance, points to an overall trend of poor blood flow perfusion in the uterine-endometrial complex. Palomba et al. (38), in their comprehensive review of endometrial function in PCOS, also emphasized the critical role of subendometrial blood flow in embryo implantation, noting that any factor affecting microcirculation in this region could directly interfere with embryo positioning and implantation. The results of this study provide hemodynamic evidence supporting this view, suggesting that increased vascular resistance from upstream sources may be an important cause of poor blood flow patterns in the downstream endometrial layer.

## 5 Study limitations

Firstly, the detection of inflammatory markers in this study was confined to several representative cytokines in the peripheral blood circulation. Direct acquisition of endometrial tissue samples for local immune cell phenotyping and cytokine profiling was not feasible. Consequently, the precise correspondence and signal transduction mechanisms between systemic inflammatory status and local immune dysregulation within the uterine microenvironment cannot be fully elucidated. Secondly, as a retrospective case-control study, this investigation could only establish correlations between inflammatory factors, metabolic parameters, and endometrial receptivity indicators, but could not infer definitive causal relationships. Future prospective cohort studies or basic experimental research are warranted to validate the molecular pathways through which inflammatory status directly leads to elevated uterine artery resistance and impaired endometrial function. Furthermore, PCOS itself exhibits significant clinical heterogeneity. Although subjects were enrolled strictly according to diagnostic criteria in this study, the lack of stratified analysis based on different phenotypes, such as the presence of obesity or typical hyperandrogenemia, may have masked potential differences in the strength of association between inflammation and endometrial dysfunction across various PCOS subgroups. This represents a significant limitation of the present study, as the pathophysiological mechanisms—and potentially the inflammatory profiles—may differ substantially between phenotypes, particularly between hyperandrogenic (A, B, C) and non−hyperandrogenic (D) subtypes. Lastly, the study population was limited to a specific geographic region and medical center. Therefore, the generalizability of the study conclusions requires further confirmation through multicenter studies with larger sample sizes.

## 6 Conclusions

This study suggests that low-grade systemic inflammation plays a pivotal role in endometrial dysfunction among patients with PCOS, potentially primarily by increasing uterine artery blood flow resistance. This finding contributes a critical immune-vascular link to the complex mechanisms underlying PCOS-related infertility, suggesting that systemic immune dysregulation, endocrine-metabolic abnormalities, and altered uterine hemodynamics intertwine to create a uterine environment unfavorable for embryonic implantation. Building upon this foundation, future research should explore whether interventions targeting inflammatory pathways, such as lifestyle modifications with anti-inflammatory effects or specific pharmacological agents, can effectively reduce uterine artery resistance, improve endometrial blood perfusion, and ultimately translate into enhanced clinical pregnancy rates for PCOS patients. Additionally, incorporating immune cell phenotyping and cytokine profiling of local endometrial tissue will be instrumental in comprehensively elucidating the specific molecular pathways from systemic inflammation to local organ dysfunction.

## Data Availability

The original contributions presented in the study are included in the article/supplementary material. Further inquiries can be directed to the corresponding author.
