# Correction: Immunological mechanisms of low-grade systemic inflammation and its role in endometrial dysfunction in women with polycystic ovary syndrome

**DOI:** 10.3389/fimmu.2026.1953539

**Published:** 2026-08-10

**Authors:** Liuhua Qu, Lu Li, Ling Yang, Ying Liu

**Affiliations:** 1 Department of Central Laboratory, The Third Affiliated Hospital of Jinzhou Medical University, Jinzhou, Liaoning, China; 2 Department of Gynaecology, Ningyuan County People’s Hospital, Yongzhou, Hunan, China

**Keywords:** anti-inflammatory cytokines, endometrial receptivity, insulin resistance, low-grade systemic inflammation, polycystic ovary syndrome, pro-inflammatory cytokines, three-dimensional ultrasound blood flow parameters

Affiliation “Department of Central Laboratory, The Third Affiliated Hospital of Jinzhou Medical University, Jinzhou, Liaoning, China” was erroneously given as “Department of Gynaecology, The Third Affiliated Hospital of Jinzhou Medical University, Jinzhou, Liaoning, China”. Also, affiliation “Department of Central Laboratory, The Third Affiliated Hospital of Jinzhou Medical University, Jinzhou, Liaoning, China” was omitted for author “Liuhua Qu”. This affiliation has now been added for author Liuhua Qu.

The original version of this article has been updated.

